# COVID-19 lockdown related to decrease in premature birth rate and increase in birth weight in metropolitan France

**DOI:** 10.3389/fped.2023.1223692

**Published:** 2023-09-07

**Authors:** Benoit Tessier, Isabella Annesi-Maesano, Gilles Cambonie, Nicolas Molinari, Nicolas Kalfa

**Affiliations:** ^1^ Department of Pediatric Surgery and Urology, Lapeyronie Hospital, CHU Montpellier—University of Montpellier, Montpellier, France; ^2^ Debrest Institute of Epidemiology and Public Health IDESP, UMR INSERM—University of Montpellier, Montpellier, France; ^3^ Department of Neonatal Medicine and Pediatric Intensive Care, Arnaud de Villeneuve Hospital, CHU Montpellier–University of Montpellier, Montpellier, France; ^4^ Department of Biostatistics and Medical Information, CHU Montpellier—University of Montpellier, Montpellier, France

**Keywords:** preterm birth, COVID-19, lockdown, birth weight, low birth weight, public health, interrupted time series analysis

## Abstract

**Introduction:**

The worldwide rate of preterm birth (PTB) has been increasing over the last two decades. COVID-19 lockdowns provide a unique opportunity to assess the effects of socioenvironmental and lifestyle factors on premature birth and birth weight. We explored the effects of COVID-19 lockdowns on the PTB rate and birth weight at a nationwide scale in France until one year after their occurrence.

**Material and Methods:**

This national retrospective observational study evaluated the rate of PTB and birth weight in France from January 2016 to December 2020. Data were obtained from the national *Programme Médicalisé des Systèmes d'Information* database. The rates of global and sub-categories of PTB were tested. The birth weight was studied before and after lockdown for all live births, for term and premature neonates, and for each category of low birth weight (LBW) by a stratified analysis.

**Results:**

Data from 2,949,372 births from January 2016 to December 2019, including 228,857 PTB, were compared to those of 699,344 births and 51,886 PTB from January to December 2020. The national rate of PTB decreased significantly from 7.7% to 7.3%, when compared with the 2016–2019 period. This decrease was persistent up to 9 months later. It was observed only for moderate PTB, whereas very PTB and extremely PTB remained stable. The national mean birth weight for full-term babies increased after the lockdown and was still observable up to 8 months later (+0.16%, *p* < 0.0001). The proportion of children with LBW also decreased 2 months after lockdown (−0.15%; *p* = 0.02). For VLBW, the difference only appeared over the 6-month post-lockdown period (−0.06%; *p* = 0.006).

**Conclusion:**

This nationwide study shows a significant reduction in prematurity and a significant increase in birth weight in France after the lockdown for a period of time not limited to the lockdown itself. A more in-depth study of the factors determining these variations may help to drive PTB prevention policies.

## Introduction

The frequency of preterm birth (PTB) varies according to the geographical area, from 5%–9% in Europe to 10.6% in North America and 11.9% in Africa (1–3). Beyond these variations, the worldwide rate of PTB has been increasing over the last two decades in most developed and developing countries (3). Every year, around 15 million children worldwide are born preterm or small for gestational age (SGA) and one million of them die (4). The consequences of this rise are serious. PTB is one of the leading causes of perinatal morbidity and mortality (5–7). It is the second cause of death in children under 5 years of age (3, 7) and the first cause of death in the first month of life. Healthcare costs for premature children are ten times more than those for children without prematurity (8). In the short term, PTB contributes to neonatal mortality (9), and later in life it also contributes to severe disabilities and morbidities in multiple developmental domains, such as hypertension and diabetes (7, 10). Unsurprisingly, the long-term consequences for public health costs are also substantial.

The exact etiopathogenesis of PTB is unknown, but it may be at the crossroads of genetic, epigenetic, biological, behavioral, environmental and maternal factors (11). Fetal growth and development are sensitive to maternal stress during pregnancy. The WHO (7) has stated that the prevention of preterm birth should be a public health priority, and understanding its underlying mechanisms is the first step toward prevention.

During the SARS-CoV-2 health crisis, three national lockdowns were decreed in France (17 March–11 May 2020; 30 October–15 December 2020; 3 April–3 May 2021). The first lockdown was the most restrictive: nearly all travel was prohibited, school and universities were closed, teleworking for all non-emergency workers became mandatory, and industrial production was stopped. Between the lockdowns, the harshness of the restrictions varied with, for example, limitations on the number of people who could gather in one place, the promotion of remote working with limited transportation, and the reduction of non-essential economic activities. Overall, these restrictions reduced the anthropogenic emission of air pollution, with possible health benefits (12–14). The COVID-19 lockdowns have thus provided a unique opportunity to assess the effects of socioenvironmental and lifestyle factors on PTB.

Previous studies have provided contrasting results. A national study in Denmark and a study in the Netherlands found a decrease in PTB compared to earlier periods (15, 16). Another study using data from London Hospital found no difference in preterm births (17) as well as in the Castilla-y-León region in Spain (18). Conversely, a study in nine Nepalese hospitals reported an increase in PTB (19). A regional study in Ireland found a decrease in the incidence of very low birth weight (20). Most of the studies were performed early after lockdown, rarely at a nationwide scale, and they typically did not report the effects on birth weight. We thus explored the effects of COVID-19 lockdowns on the PTB rate and birth weight at a nationwide scale in France until one year after their occurrence.

## Material and methods

This national retrospective observational study evaluated the evolution of the rate of PTB and birth weight in all of metropolitan France from January 2016 to December 2020, particularly focusing on the effects of the first and most restrictive lockdown from 16 March to 11 May, 2020. Data were obtained from the exhaustive standardized data of the national *Programme Médicalisé des Systèmes d'Information* (PMSI) database, which contains medical-economic information on admissions to French public and private hospitals, based on the International Classification of Diseases (ICD-10).

PTB categories were defined according to the WHO definition as a live birth before 37 weeks of gestation, with subcategories as follows: moderate PTB: from 32 to 36 + 6 weeks of gestation, very PTB: from 27 + 6 to 31 + 6 weeks of gestation, and extremely PTB: before 28 weeks of gestation. Birth weight was defined as follows: low birth weight: <2,500 g, very low birth weight: <1,500 g, and extremely low birth weight: <1,000 g.

A comparative analysis between the rate of PTB during the 2016–2019 period and the lockdown period was performed using a score test. We tested various periods from the lockdown itself to the whole year following it. Each comparison was based on similar months of previous years to avoid bias due to seasonal variations. Global PTB and the PTB subcategories were tested separately. We then studied the birth weight before and after lockdown at three levels: birth weight of all live births, separate analyses for term and premature neonates (Student t-test analysis), and stratified analyses for low, very low and extremely low birth weight (score test analysis). Statistical analysis was performed using R (version 4.0.3) software.

## Results

Overall, 3,648,716 live births were included between January 2016 and December 2020, out of a population of 66.99 million inhabitants. Data from 2,949,372 births from January 2016 to December 2019, including 228,857 PTB, were compared to those of 699,344 births and 51,886 PTB from January to December 2020 (Table 1).

**Table 1:** Comparison of prematurity lockdown and previous years (mismatches with the total number of infants is due to missing data for birth weight in less than 0.01% of the cohort) (period of lockdown are grayed out).

Years	Live birth tot	Prematurity	Moderate PTB	Very PTB	Extremely PTB
2016	760,299	59,619 (7.8%)	47,696 (6.3%)	11,923 (1.6%)	5,728 (0.7%)
2017	744,806	58,113 (7.8%)	46,092 (6.2%)	12,021 (1.6%)	5,978 (0.8%)
2018	729,199	56,785 (7.8%)	45,032 (6.2%)	11,753 (1.6%)	5,699 (0.8%)
2019	715,068	54,340 (7.6%)	43,064 (6%)	11,276 (1.6%)	5,404 (0.8%)
2020	699,344	51,886 (7.4%)	40,902 (5.8%)	10,984 (1.6%)	5,324 (0.8%)
Before and after lockdown					
Before lockdown	29,49,372	228,857 (7.8%)	181,884 (6.2%)	46,973 (1.6%)	22,809 (0.8%)
During and after lockdown	699,344	51,886 (7.4%)	40,902 (5.8%)	10,984 (1.6%)	5,324 (0.8%)

**Figure 1:**
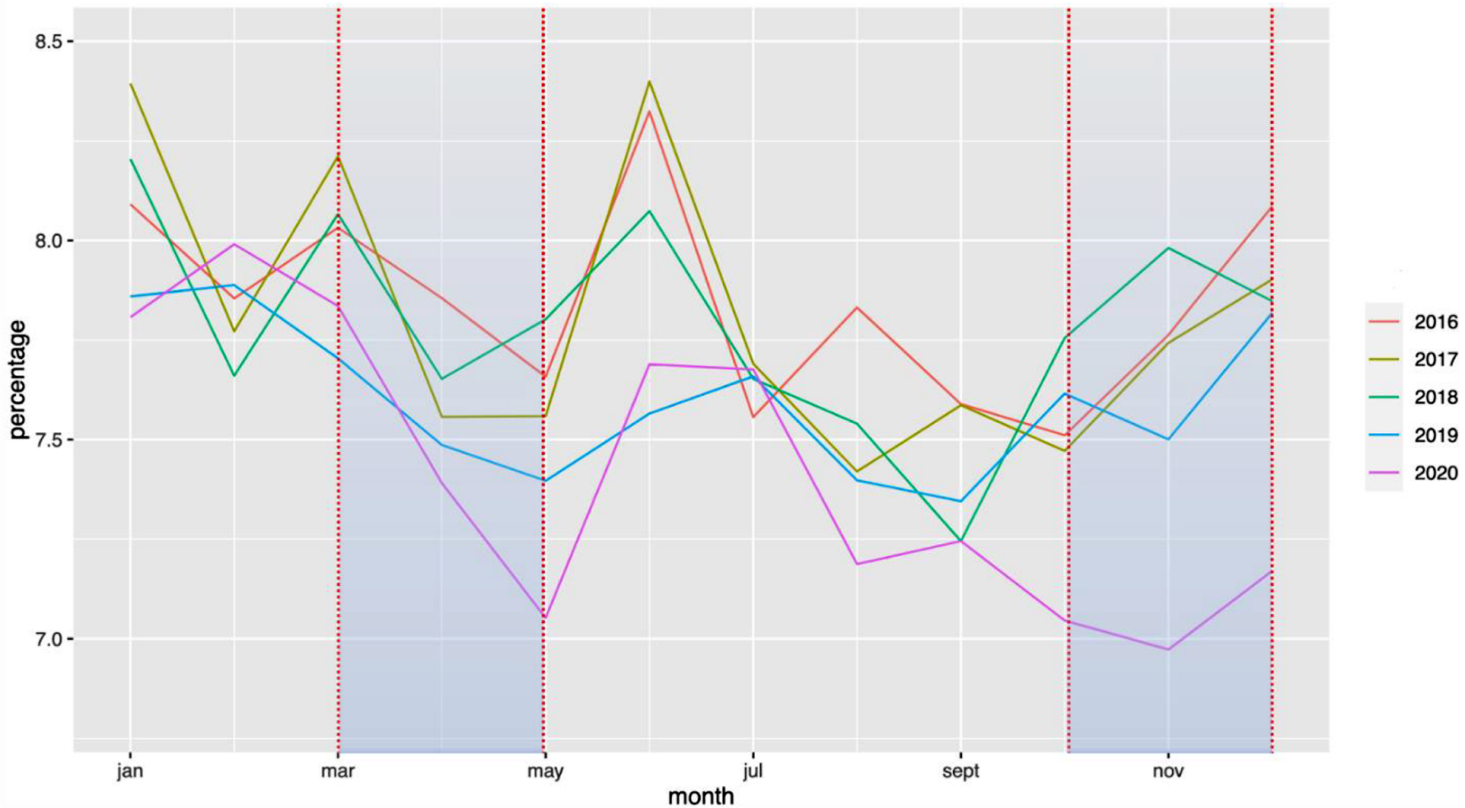
Change in the rate of premature births between January 2016 and December 2020. The rate of PTB is expressed in percentage of all deliveries. Each color represents one year.

**Figure 2:**
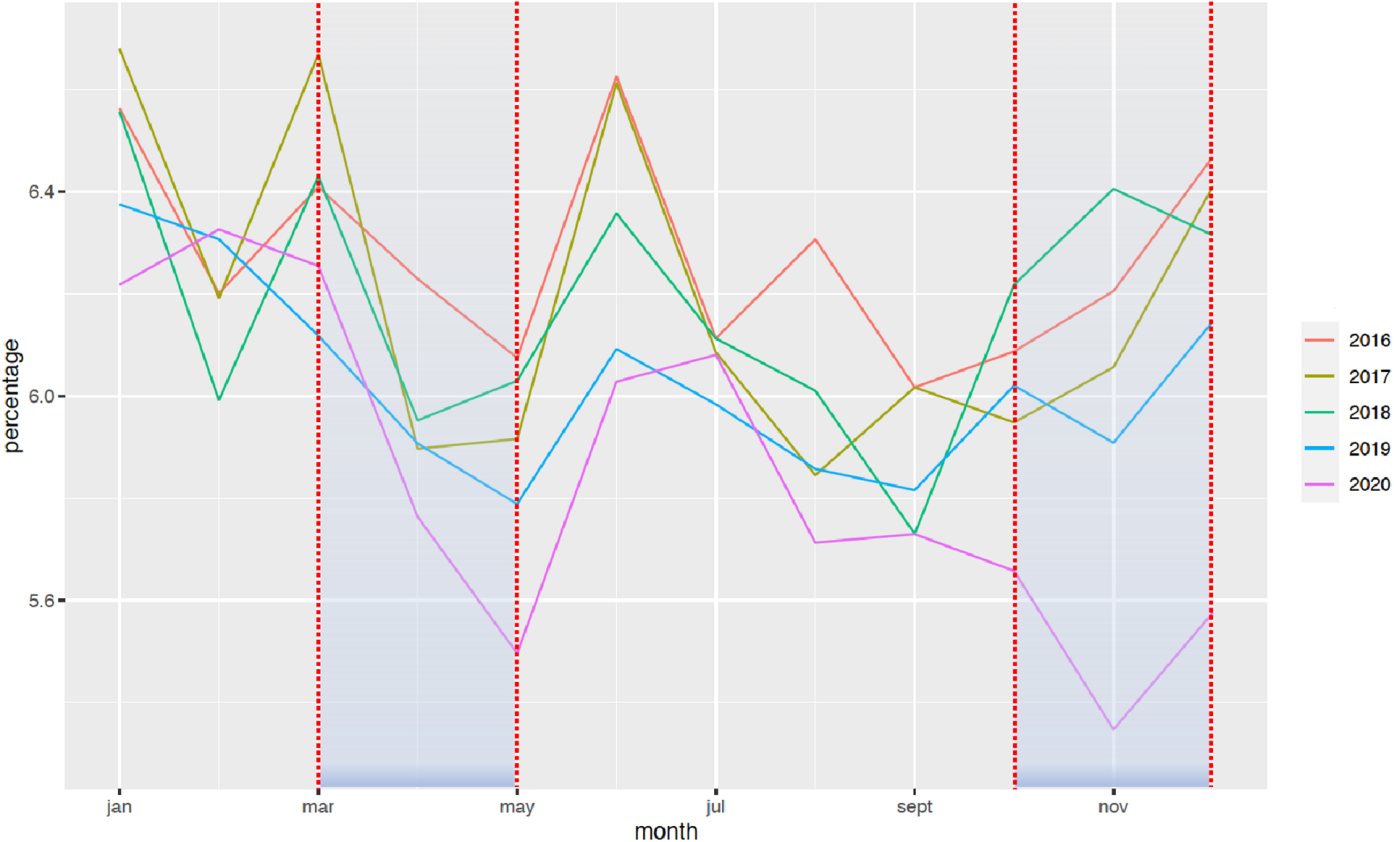
Change in the rate of moderate premature births between January 2016 and December 2020. The rate of PTB is expressed in percentage of all deliveries. Each color represents one year.

**Figure 3:**
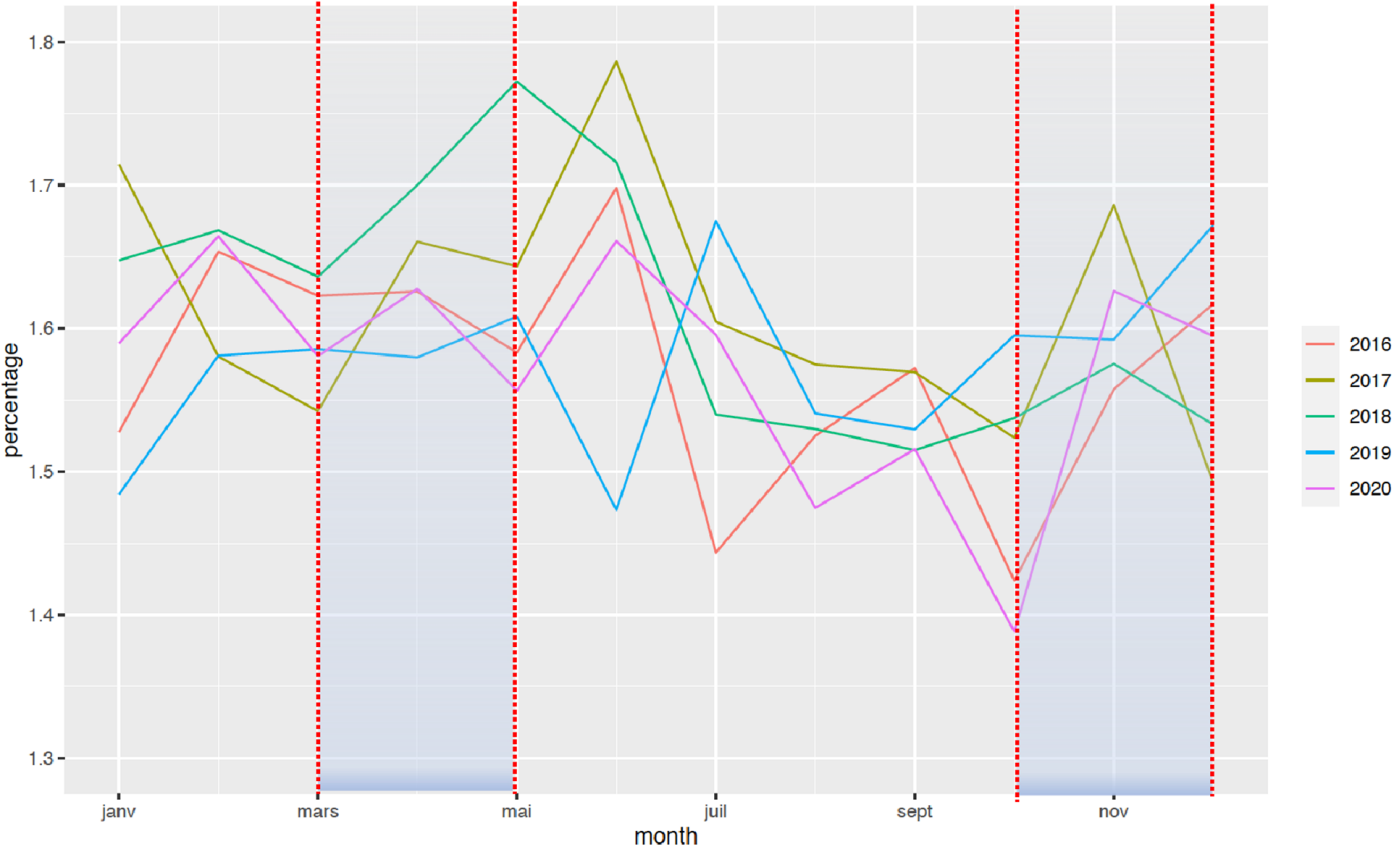
Change in the rate of very PTB between January 2016 and December 2020. The rate of PTB is expressed in percentage of all deliveries. Each color represents one year.

**Figure 4:**
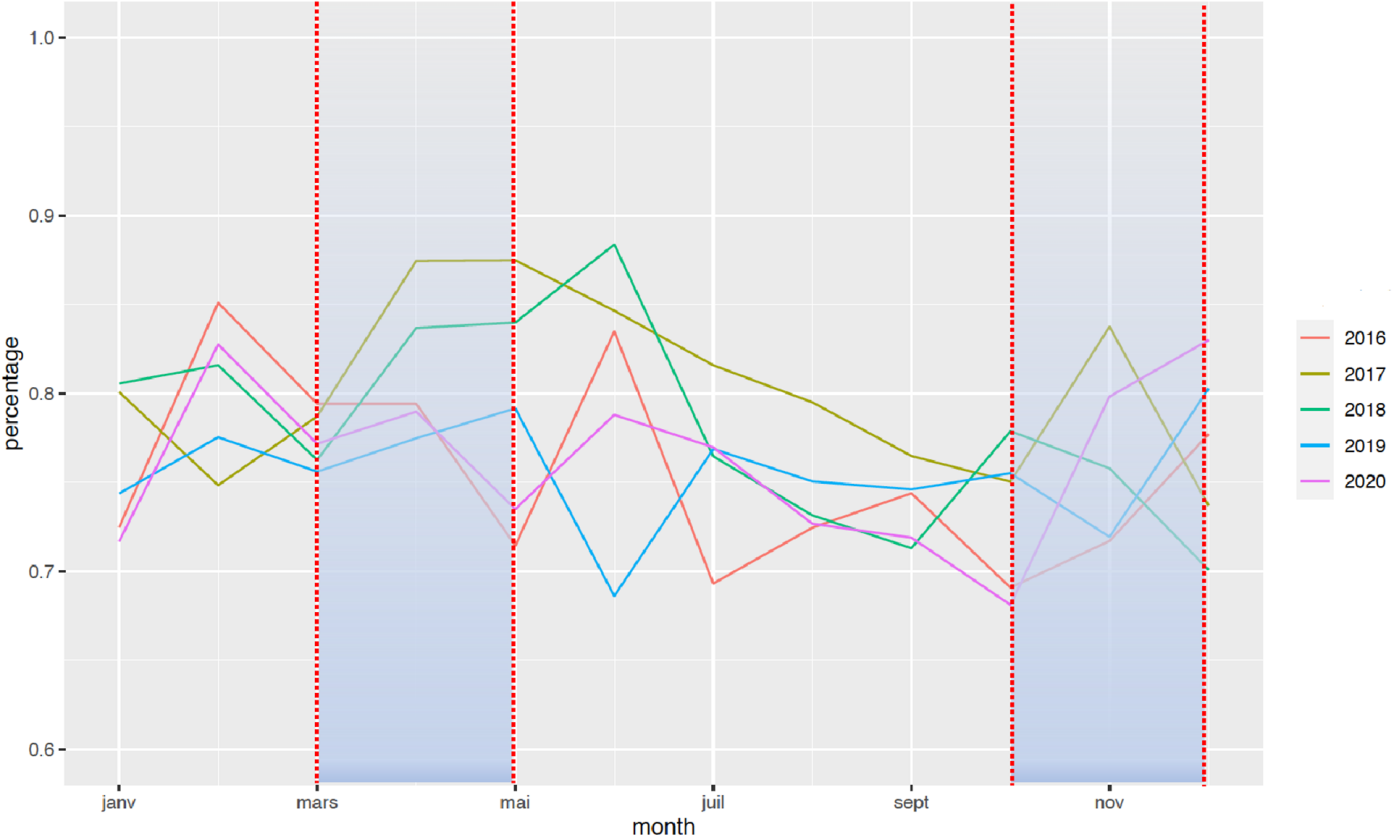
Change in the rate of extremely PTB between January 2016 and December 2020. The rate of PTB is expressed in percentage of all deliveries. Each color represents one year.

The national rate of PTB from March to December 2020 decreased significantly from 7.7% to 7.3%, when compared with the 2016–2019 period (Figure 1). Two points are relevant regarding the timing of this reduction and the type of PTB. First, the decrease in the timing was observable early on, from the first 2 months of lockdown [March–April, RR = 0.97 (0.99–0.95)], and this decrease was persistent up to 9 months later (March–August, RR = 0.96 [0.97–0.95]; March–December, RR = 0.95 [0.96–0.94]). Regarding the type of PTB, this decrease was observed only for moderate PTB [March–July, RR = 0.997 (0.998–0.996)], whereas very PTB and extremely PTB remained stable at that time (very PTB: March–July, RR = 0.98 [1.02–0.96]; extremely PTB: March–July, RR = 0.97 [1.01–0.92]) (Figures 2–4).

**Figure 5:**
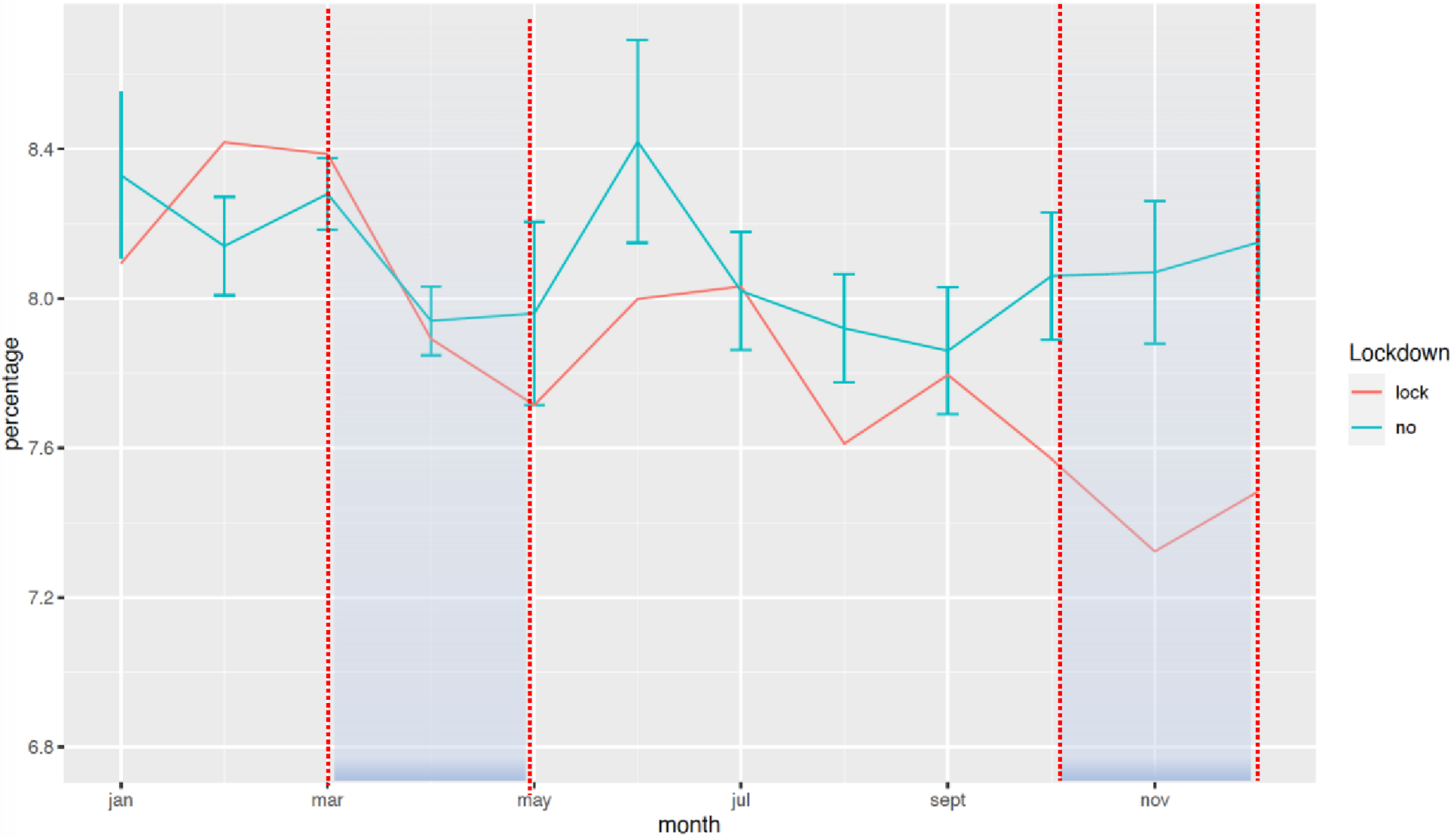
Change in the rate of LBW between January 2016 and December 2020. The rate of LBW is expressed in percentage of all deliveries. The red curve represents the mean of all years prior to 2020 with standard deviation, and the blue curve represents the year of lockdown.

The national mean birth weight for full-term babies increased after the lockdown (3,327 g vs. 3,332 g, +0.16%, *p* < 0.0001). This increase was detectable 2 months after the start of lockdown (3,328 g vs. 3,332 g, +0.11%; *p* = 0.003) and was observable up to 8 months later. To further explain this increase in birth weight in full-term babies, we explored the evolution of gestational age and the proportion of SGA infants among these full-term infants. We found no significant difference in the gestational age in full-term infants before (39.3 weeks of gestation) and during and after lockdown (39.3 weeks of gestation). Regarding the rate of SGA, it decreased from 8.98% before lockdown to 8.73% during and after lockdown (*p* = 3.10–10) (Table 2). This observation was strictly limited to the 2020 period and there was no such tendency in previous years (Table 3). The proportion of children with LBW also varied after the lockdown, with a decreased incidence 2 months post-lockdown (−0.15%; *p* = 0.02) (Figure 5). For VLBW, the difference only appeared over the 6-month post-lockdown period (−0.06%; *p* = 0.006). For ELBW, there was no difference between the lockdown period and previous years. This increase in birth weight was also observable for premature babies (2,094 g vs. 2,078 g, *p* = 0.03) (Table 4).

**Table 2:** Comparison of small for gestational age in full term babies between lockdown and previous years (period of lockdown are grayed out).

		SGA	No SGA	Total
37 weeks of gestation	No lockdown	18,246 (9.8%)	168,729	186,975
	Lockdown	4,439 (10.1%)	39,577	44,016
38 weeks of gestation	No lockdown	42,274 (9.6%)	409,825	452,099
	Lockdown	9,643 (8.9%)	99,141	108,784
39 weeks of gestation	No lockdown	74,651 (9.3%)	724,906	799,557
	Lockdown	17,408 (8,9%)	176,601	194,009
40 weeks of gestation	No lockdown	69,173 (9.1%)	689,835	759,008
	Lockdown	15,832 (8.9%)	161,432	177,264
41 weeks of gestation	No lockdown	39,937 (7.6%)	482,273	522,210
	Lockdown	9,216 (7.5%)	114,063	123,279
Total	No lockdown	244,281 (8.9%)	24,75,568	27,19,849
	Lockdown	56,538 (8.7%)	590,814	647,352

**Table 3:** Comparison of small for gestational age in full term babies between each years (period of lockdown are grayed out).

	2016		2017		2018		2019		2020	
	SGA	No SGA	SGA	No SGA	SGA	No SGA	SGA	No SGA	SGA	No SGA
37 weeks of gestation	4,656	44,267	4,505	42,340	4,467	41,329	4,618	40,793	4,439	39,577
38 weeks of gestation	10,916	106,862	10,793	102,761	10,449	100,773	10,116	99,429	9,643	99,141
39 weeks of gestation	19,219	188,891	18,662	181,784	18,802	177,832	17,968	176,399	17,408	176,601
40 weeks of gestation	17,649	178,344	17,364	175,043	17,601	169,938	16,559	166,510	15,832	161,432
41 weeks of gestation	9,840	119,832	10,222	123,077	10,130	120,918	9,745	118,446	9,216	114,063
Total	62,280 (8.9%)	638,196	61,546 (9.0%)	625,005	61,449 (9.1%)	610,790	59,006 (8.9%)	601,577	56,538 (8.7%)	590,814

**Table 4:** Comparison of low birthweight, very low birth weight, extremely low birth weight and birthweight for full term babies and preterm birth between lockdown and previous years (period of lockdown are grayed out).

Time period	Lockdown	<2,500 g	>2,500 g	RR [95% IC]	<1,500	RR [95% IC]	<1,000 g	RR [95% IC]
March–April	No	37,792	428,069	0.995 [0.974- 1.017]	7,520	0.99 [0.948–1.049]	4,112	1.013 [0.945–1.085]
	Yes	9,212	103,818		1,830		985	
March–May	No	57,511	656,074	1.007 [0.989–1.025]	11,595	1.021 [0.979–1.064]	6,259	1.017 [0.961–1.075]
	Yes	13,757	158,187		2,737		1,484	
March–June	No	78,086	879,714	1.019 [1.003–1.035]	15,708	1.02 [0.98–1.058]	8,424	1.02 [0.971–1.07]
	Yes	18,457	212,247		3,706		1,990	
March–July	No	99,393	11,24,024	1.015 [1.001–1.029]	19,849	1.012 [0.981–1.045]	10,619	1.007 [0.964–1.051]
	Yes	23,466	269,600		4,697		2,526	
March–August	No	120,043	13,64,161	1.019 [1.006–1.032]	23,939	1.022 [0.993–1.052]	12,778	1.015 [0.975–1.056]
	Yes	28,137	326,297		5,592		3,007	
March–September	No	139,707	15,94,733	1.017 [1.006–1.029]	27,792	1.024 [0.997–1.052]	14,793	1.016 [0.98–1.055]
	Yes	32,745	380,804		6,473		3,470	
March–October	No	160,601	832,944	1.023 [1.012–1.034]	31,833	1.035 [1.01- 1.062]	16,931	1.028 [0.993–1.064]
	Yes	37,402	437,658		7,326		3,925	
March–November	No	180,170	20,55,837	1.031 [1.021–1.042]	35,761	1.03 [1.006–1.006]	18,987	1.019 [0.986–1.053]
	Yes	41,577	490,489		8,262		4,433	
March–December	No	200,142	22,80,864	1.036 [1.026–1.047]	39,602	1.027 [1.004–1.05]	21,005	1.009 [0.979–1.041]
	Yes	45,707	5,41,542		9,129		4,926	

Time period	Lockdown	Birth weight for full term (g)	*P* value	Birth weight for PTB (g)	*P* value
March–April	No	3,326	0.119	2,099	0.11
	Yes	3,328		2,085	
March–May	No	3,328	0.003	2,095	0.03
	Yes	3,332		2,079	
March–June	No	3,328	5.963 × 10^−6^	2,096	0.039
	Yes	3,334		2,083	
March–July	No	3,328	2.665 × 10^−7^	2,098	0.021
	Yes	3,333		2,085	
March–August	No	3,328	2.599 × 10^−6^	2,099	0.16
	Yes	3,332		2,091	
March–September	No	3,328	5.12 × 10^−7^	2,099	0.09
	Yes	3,332		2,091	
March–October	No	3,327	4.873 × 10^−8^	2,099	0.15
	Yes	3,332		2,093	
March–November	No	3,328	1.965 × 10^−12^	2,099	0.017
	Yes	3,333		2,089	
March–December	No	3,327	6.024 × 10^−15^	2,101	0.005
	Yes	3,333		2,090	

To evaluate a particular pattern of these changes in metropolitan cities, additional analyses were carried out in the three largest cities of France: Paris, Lyon and Marseille. No difference was found for overall prematurity or mean weight in full-term infants in these areas. The increase in the birth weight of preterm infants was observed only during lockdown.

## Discussion

The study found that, at the nationwide scale, the COVID-19 lockdown was associated with a reduction in PTB beyond the time of lockdown and a concomitant increase in mean birth weight. Regarding prematurity, these data are consistent with previous studies. For instance, a national study in the Netherlands found a decrease in the incidence of PTB across various time windows around the implementation of COVID-19 mitigation measures (16). A study in Denmark found a significant decrease in PTB during the lockdown period compared with the previous 5 years (15). In France, a similar decrease in PTB was identified during the first lockdown with a shorter follow-up (21). Other groups did not find similar results (17, 19), but these discrepancies may be explained by several factors, such as a focus on specific populations, with different ethnicities and different genetic backgrounds; differences across the health systems; or examination of a particular geographical area rather than at the scale of a whole nation.

Interestingly, the lockdown had differential effects on PTB according to the severity of the prematurity. In France, we and others (21) have shown that the reduction was mainly observed for moderate PTB, whereas very and extremely PTB were not affected. This may be explained by differences in the pathophysiological mechanisms for moderate and severe prematurity. Severe prematurity is usually associated with multiple pregnancies (twins/triplets), vascular dysfunction of the placenta (hypertension, pregnancy toxemia/preeclampsia: HELLP syndrome), premature rupture of membranes, chorioamnionitis, and major congenital birth defects (22). Moderate prematurity remains multifactorial, in most cases related to various risk factors at the end of pregnancy such as excessive physical activity, dietary factors, exposure to air pollution, or an excessive workload (23–26). These factors might have been modified by the lockdowns, as they imposed radical changes in lifestyle behaviors. A decrease in physical activity, a reduction in commuting times, and changes in dietary habits were observed at that time (27, 28). Variations in air pollution levels were also observed for some compounds, but the levels differed according to the substance (12, 13, 29, 30). Lockdowns thus offer new insights into the pathophysiology of moderate prematurity and point toward actionable parameters to prevent it. PTB is increasing in France (6.5% vs. 7.5% in 2010 and 2016, respectively) as well as worldwide. It is responsible for significant health costs (8), morbidity (6, 7) and neonatal mortality (5). In France, almost a quarter of neonatal deaths (24.4%) occur in the first day of life and half (47.8%) in the first week after birth. Although infant mortality has several causes and the rate of neonatal deaths remains stable at the moment (31), prematurity and birth weight play major roles (9). These observations from the COVID-19 lockdown support the idea that moderate PTB may be preventable. The next step will be to determine which actions public authorities are willing to take to prevent PTB-related morbidities and drive down health costs.

A trend toward a decrease in PTB was observed in 2019 compared to previous years (7.55% vs. 7.77% *p* = 0.001). This trend then accelerated after lockdown (7.32%). Our results also showed that the reduction in prematurity was not limited to the initial period of lockdown and persisted for at least up to 9 months post-lockdown. Several factors may explain this lasting mid-term effect: the gradual lifting of the measures, the continuation of teleworking for most people, the protection of pregnant women against other viral infections, and the long-lasting effects of the lockdown measures at the start of pregnancy on later placenta function.

To further confirm the effect of the lockdown on fetal maturation and growth, we explored the data on birth weight in France. We reported an early reduction in the incidence of LBW and a later reduction in VLBW with no significant effect on ELBW. The effects of lockdowns on LBW have also been suspected by others (20, 32), but the data on VLBW are quite conflicting, ranging from no effects (33) to strong variations (20). VLBW represents only a small part of births (1.5%) (34) and the limited size of the series compared to a whole population may explain these discrepancies. Interestingly, the weight of full-term babies also increased significantly in France. This increase in birth weight for full-term babies was not explained by an increase in their gestational age but rather by a decreased rate of SGA babies during and after lockdown. This change is all the more relevant in that the rate of SGA did not tend to decrease in the previous 4 years. The difference of the mean birthweight before and after lockdown remains limited and probably does not influence the clinical management of the neonate at the individual level. But this data might be still relevant to reflect the influence of lockdown on the fetal growth as suggested by a more visible difference on the 5th and on the 10th percentiles (2,270 g vs. 2,290 g for the 5th percentile, 2,580 vs. 2,600 g for the 10th percentile). This supports the idea that the lockdown had a beneficial effect not only on PTB babies, but on the whole population and all pregnant women. These results are in accordance with the previously described differences in birth weight associated with the 2008 Beijing Olympics air pollution reduction and demonstrate it at the level of a whole country. Like prematurity, birth weight is a relevant parameter for public health. LBW is associated with hypertension, sleep-disordered breathing, kidney disease, neurodevelopmental and psychiatric disorders, and metabolic risk (35, 36). SGA is a risk factor for a number of diseases from the neonatal period to adulthood. The mortality of neonates with SGA is higher than of those without SGA. During childhood and adolescence, SGA increases the risk of growth retardation, neurodevelopment delay, decreased gonadal function and metabolic disorders such as dyslipidemia, hypertension, diabetes and obesity. The risk of cardiovascular disease and cancer is also increased in adult life (37, 38). The COVID-19 lockdown may point toward actionable parameters to prevent LBW and SGA. The exact role of each of these factors (occupational hazards, environmental exposures, daily commuting, remote working …) remains to be determined.

It remains unclear why the variations observed at the national level were largely unobservable in France’s three main metropolitan cities. Urban migration has been described during the lockdowns, in France as in other countries, which may have had a variable impact on the rate of prematurity, depending on the characteristics of the population that left these areas (39). These data may also suggest a difference in the effects of lockdown, depending on the environment and lifestyle of the pregnant women. For instance, commuting distances are longer in rural areas, and lockdown may have had a greater impact in these parts of the country. Data from metropolitan cities across the world also show very different patterns of PTB variations. For instance, in a paper reporting 3 cities in Canada and China, the change of PTB rate during lockdown was highly heterogeneous, either non-significant (Shenzen), or limited to very and extremely PTB (Calgary) or limited to moderate PTB (Edmonton) (40). More precise subgroup studies, including geographic coding, are the next step.

Some limitations should be acknowledged, but they are inherent to observational and retrospective studies at a country scale. First, daily living habits changed during the lockdown, but details about these changes were not accessible. For instance, the diets of pregnant women were probably different (28), as was the consumption of tobacco, alcohol and drugs (41), but individual data are lacking to directly link a specific factor to the reduction in PTB or the increase in birth weight. Second, the lockdown was reinstituted in a sequential manner with different restrictions each time. Determining the role of each restriction was thus not possible. A second limitation of this study is that aggregated data in ecological studies can introduce bias, including aggregation bias and association bias. We cannot exclude aggregation bias due to data grouped at the group level that do not consider individual-level variations. Similarly, due to association bias, the erroneous assumption that the associations observed at the group level apply to the individuals, without adjusting or potential confounders, within the group is possible. Another limitation is the absence of data on stillbirths. Last, the announcement of the pandemic and the lockdown created a psychologically stressful event in the general population (42), and this may have interfered with the effects of the lockdown on PTB and birth weight.

Overall, this nationwide study shows a significant reduction in prematurity and a significant increase in birth weight in France after the lockdown for a period of time not limited to the lockdown itself. A more in-depth study of the factors determining these variations may help to drive PTB prevention policies.

## Data Availability

The raw data supporting the conclusions of this article will be made available by the authors, without undue reservation.
